# Effects of Virtual Reality Exercises on Chronic Low Back Pain: Quasi-Experimental Study

**DOI:** 10.2196/43985

**Published:** 2023-09-15

**Authors:** M Waqar Afzal, Ashfaq Ahmad, Hafiz Muhammad Bilal Hanif, Nauman Chaudhary, Syed Amir Gilani

**Affiliations:** 1 Department of Physical Therapy University of Lahore Lahore Pakistan; 2 Department of Public Health Institute of Social & Cultural Studies University of the Punjab Lahore Pakistan; 3 Faculty of Allied Health Sciences University of Lahore Lahore Pakistan

**Keywords:** low back pain, lumbar range of motion, pain, Oswestry disability index, virtual reality, exercise, back pain, lumbar, range of motion, virtual reality, VR, rehabilitation, gaming, serious game

## Abstract

**Background:**

Low back pain is a common health problem globally. Based on the duration of pain, it is classified as acute, subacute, or chronic low back pain. Different treatment strategies are available to reduce chronic low back pain. Virtual reality (VR) is a novel approach in back pain rehabilitation.

**Objective:**

This study aimed to compare the effects of VR games on chronic low back pain.

**Methods:**

This quasi-experimental study was conducted among 40 patients with chronic low back pain. The data were collected using a nonprobability, convenient sampling technique. Patients visiting the Department of Physiotherapy, Government Services Hospital, Lahore, Pakistan, were recruited and equally divided into 4 groups. Group A received the Reflex Ridge game; group B received the Body Ball game; group C combined the 2 games without back-strengthening exercises; and group D combined the 2 games with back-strengthening exercises. The participants received 8 treatment sessions, with 3 sessions/wk. The outcomes were pre- and posttest measurements of pain intensity, low back disability, and lumbar range of motion. The repeated measurement ANOVA was used for inter- and intragroup comparison, with significance at *P*≤.05.

**Results:**

The study comprised a sample of 40 patients with low back pain; 12 (40%) were female and 28 (60%) were male, with a mean age of 37.85 (SD 12.15) years. The pre- and posttest mean pain scores were 7.60 (SD 1.84) and 4.20 (SD 1.62) in group A, 6.60 (SD 1.776) and 5.90 (SD 1.73) in group B, 6.90 (SD 1.73) and 5.40 (SD 1.07) in group C, and 7.10 (SD 1.53) and 3.60 (SD 0.97) in group D, respectively. The mean pain score differences of group D (combining the Reflex Ridge and Body Ball games with back-strengthening exercises) compared to groups A, B, and C were –.60 (*P*=.76), –2.30 (*P*<.001), and –1.80 (*P*=.03), respectively. Regarding the range of motion, the forward lumbar flexion mean differences of group D compared to groups A, B, and C were 3.80 (*P*=.21), 4.80 (*P*=.07), and 7.40 (*P*<.001), respectively. Similarly, the right lateral lumbar flexion mean differences of group D compared to groups A, B, and C were 2.80 (*P*=.04), 5.20 (*P*<.001), and 4.80 (*P*<.001), respectively. The left lateral lumbar flexion mean differences of group D compared to groups A, B, and C were 2.80 (*P*<.001), 4.80 (*P*=.02), and 2.20 (*P*<.001). respectively, showing significant pre- and posttreatment effects.

**Conclusions:**

VR exercises had statistically significant effects on improving pain, low back disability, and range of motion in all groups, but the combination of Reflex Ridge and Body Ball games with back-strengthening exercises had dominant effects compared to the other groups.

**Trial Registration:**

Iranian Registry of Clinical Trial IRCT20200330046895N1; https://en.irct.ir/trial/46916

## Introduction

Low back pain (LBP) is a prevalent health concern that becomes more common as people age [1]. Based on the duration of pain, it is further classified into 3 categories: acute, subacute, or chronic LBP [2]. LBP affects people of all ages, from children to older adults, and can afflict people in high-, middle-, and low-income countries. People with physically demanding jobs, those with physical and mental illnesses, smokers, and people with obesity are more likely to have LBP [3]. From the third decade of life to 60 years of age, the frequency of chronic LBP rises linearly, with women being more affected [4]. Furthermore, the fear of pain is more strongly linked to impairment in people with chronic LBP than in people with acute LBP [5]. Patients avoid spinal flexion because of the fear of pain, especially lumbar flexion [6]. This kinesiophobia can be managed using virtual reality (VR) maneuvers including the neuromodulation of body perception, distraction, and graded exposure therapy. These 3 mechanisms are considered the theoretical basis of VR therapeutic effects [7].

To treat persistent LBP, various treatments, including nonpharmacological interventions, can be used. VR is a type of rehabilitation technology that allows users to engage in a computer-generated environment [8]. Recently, the development of portable and affordable motion tracking systems has broadened the use of VR in the management and rehabilitation of patients with musculoskeletal pain [9]. VR has 3 elements: interaction, immersion (sometimes nonimmersive), and imagination [10]. Through a head-mounted display that follows the movement of the participant’s body, VR gives the sensation of being entirely encircled by a virtual world [11]. VR games have already been integrated into rehabilitation programs for patients with chronic pain [12]. Distraction is one of the suggested mechanisms that explains the effects of VR on pain [13]. In orthopedic rehabilitation, clinical trials have previously assessed VR effectiveness in individuals with different musculoskeletal disorders, including ankle sprain, anterior cruciate ligament injury, and frozen shoulder [14]. It also makes it possible to increase movement in patients with kinesiophobia due to chronic pain [15]. Among different treatment regimens, the use of isokinetic and VR exercises is considered to be effective [16]. The idea is to catch the attention of the user in such a way that the patient’s mind focuses on the game while performing game tasks that are actually exercises for pain and rehabilitation [17]. With this approach, we are able to translate clinical guidelines into the VR environment to facilitate future implementation in the care pathway [18]. Virtual exercises are based on body movements including catching, squatting, bending, jumping, and a combination of these movements during the rehabilitation process [19]. The lack of adequate physical activity or sedentary lifestyle is one of the major problems [20]. The use of virtual embodiment to influence body perception is beginning to receive more attention, and it might have clinical implications for disorders such as chronic pain that include altered body image [21].

Various studies have reported the positive impact of VR games, but there is a need to explore the comparison and combination of different routine VR games. Different VR games can help manage chronic LBP. This study aimed to assess the effects of 2 games, the Reflex Ridge and Body Ball VR games, in patients with chronic LBP. We hypothesized that the VR games used would constitute an acceptable exercise program for patients with chronic LBP.

## Methods

### Design

This was a quasi-experimental study conducted in Lahore, Pakistan. Initially, a randomized controlled trial had been intended; however, due to the COVID-19 pandemic and the uncertainties caused by it, the study design was changed to a quasi-experimental study. The institutional review board of the University of Lahore approved the amendments made to the research project.

### Ethical Considerations

The ethical approval of the study was obtained from The University of Lahore (IRB-UOL-FAHS/696-IV/2020).

### Participants and Settings

A total of 70 patients with LBP were screened for the study, and 40 participants (10 in each group) were recruited from the Department of Physiotherapy, Government Services Hospital, Lahore, Pakistan. The participants were recruited using the nonprobability, convenient sampling technique.

### Sample Size

The sample size was calculated as follows:




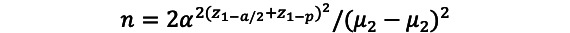




where z_1 – α/2_ was the level of significance, µ_1_ was the expected mean of the visual analogue scale (4.0) in group A [22], µ_2_ was expected mean of the visual analogue scale (5.0) in group B [22], the expected SD was 0.75 in group A and 2.0 in group B, the power of study was 80%, and the expected sample size was 40 (n=10 for each group).

### Patient Characteristics

Basic information regarding age, BMI, marital status, occupation, and symptoms with a complete history was obtained before enrollment.

### Interventions and Procedures

Pretest assessment was made after informed consent from all participants. In all, 40 patients with chronic LBP were equally divided into 4 groups following the inclusion and exclusion criteria (Textbox 1).

**Study selection criteria of patients with low back pain.**

**Inclusion criteria**

- All genders
- Aged 25-50 years
- Low back pain that lasted more than 12 weeks
- Nonradiating pain

**Exclusion criteria**

- Recurrent low back pain
- Neurological symptoms
- Any previous history of fracture in the spine or lower limb, cardiac or endocrine disease, or neurological disorders such as Parkinson disease and stroke

Group A was given the Reflex Ridge game, and group B was given the Body Ball game. Group C combined the Reflex Ridge game with the Body Ball game without back-strengthening exercises; the rest of treatment protocol was same. Group D combined the Reflex Ridge game with the Body Ball game along with back-strengthening exercises, including bridging, prone leg raises, trunk extension while keeping the arms on the back, and trunk rotation exercises [23]. After the VR exercises, all groups were given moist heat therapy with transcutaneous electrical nerve stimulation for 10 minutes, with a frequency of 10 repetitions. The participants received 8 treatment sessions, with 3 sessions/wk.

VR was provided through the Kinect Xbox 360 device (v.2 model; Microsoft) [24]. This sensitive device for motion sensing incorporates time-of-flight and red-green-blue cameras for the detection of body skeletal movements and real-time gesture evaluation. This is attached to an LCD monitor. In the Reflex Ridge game, participants performed different movements (lumbar side bending, lumbar movement with shoulder elevation, sitting, and jumping) to avoid hitting the obstacle. In the Body Ball game, arm and leg movements were used to hit the ball.

### Outcomes

The outcome measures were pain intensity, low back disability, and lumbar range of motion (ROM), measured through a numerical pain rating scale, the Oswestry disability index (ODI), and pre- and posttest evaluations.

#### Pain Intensity

Pain intensity was measured using the numerical pain rating scale. Patients were asked to select a circle that best describes the current level of pain, from 0 (no pain) to 10 (severe pain) [25].

#### Low Back Disability

Low back disability was measured using the ODI. It is considered valid and suitable for the assessment of disability among patients with LBP [26]. It consists of 10 sections including pain, personal care, sitting, lifting, walking, sleeping, standing, traveling, social life, and sexual life, each having scores from 0-5 with a total score of 50. It is a broader level assessment of disability compared to pain intensity alone [27].

#### Lumbar ROM

Lumbar ROM was recorded using a gravity-based inclinometer in the standing position. The inclinometer was placed at the T12-L1 level of the spinal column, marked, and zeroed. Flexion and extension were measured at the T12-L1 level with a command to bend forward and backward, respectively. The right and left lumbar lateral flexion ROMs were measured by keeping the inclinometer parallel to the axis of the spinal column, and patients were asked to bend on their respective sides with fingertips pointed down toward the respective side of the thigh [28]. The inclinometer has a good reliability for measuring spinal (*r*=0.97), flexion (*r*=0.98), and extension (*r*=0.75) ROMs [29].

### Data Analysis

All data were encoded and entered anonymously and remained confidential. IBM SPSS (version 24.0) was used for statistical analysis. The means and SDs of quantitative data were measured. However, frequencies and percentages were used to present categorical data. Normality tests were applied for data distribution using skewness, kurtosis, and the Shapiro-Wilks test. The distribution of data was normal as the *P* value was >.05. For pre- and posttest evaluations, parametric repeated measurement ANOVA was used to analyze intragroup comparisons and measure mean intergroup differences for pain intensity, low back disability, and lumbar ROM. The tests were conducted at a significance level of *P*≤.05.

## Results

### Patient Characteristics

The study comprised 40 patients with LBP; 12 (40%) were female and 28 (60%) were male, with a mean age of 37.85 (SD 12.15) years. All the participants were married. Most had a BMI in the normal (n=13, 32%) and overweight (n=18, 45%) categories (Table 1).

**Table 1:** Patient characteristics.

Demographics and category		Value (n=40), n (%)
**Sex**		
	Male	28 (60)
	Female	12 (40)
**BMI**		
	Underweight	6 (15)
	Normal	13 (32)
	Overweight	18 (45)
	Obese	3 (8)
**Occupation**		
	Teacher	14 (35)
	Shopkeeper	16 (40)
	Computer worker	8 (20)
	Banker	2 (5)

### Outcomes

#### Pain Intensity

The pre- and posttest mean pain scores were 7.60 (SD 1.84) and 4.20 (SD 1.62) in group A, 6.60 (SD 1.776) and 5.90 (SD 1.73) in group B, 6.90 (SD 1.73) and 5.40 (SD 1.07) in group C, and 7.10 (SD 1.53) and 3.60 (SD 0.97) in group D, respectively (Table 2). The mean pain score differences of group D compared to groups A, B, and C were –.60 (*P*=.76), –2.30 (*P*<.001), and –1.80 (*P*=.03), respectively (Table 3). There was a significant improvement in pain rating in all groups (all *P*<.05), but pre- and posttest differences showed a significant improvement in group D for pain (*P*<.001).

**Table 2:** Intragroup comparison for pain and disability index.

Group and evaluation		Pain rating				ODI^a^				
		Mean (SD)	Mean SE	*P* value^b^	Mean (SD)		Mean SE		*P* value^b^	
**Group A**				.001						<.001
	Pretest	7.60 (1.84)	.58		25.10 (3.035)		.959			
	Posttest	4.20 (1.62)	.51		13.10 (1.85)		.586			
**Group B**				.02						<.001
	Pretest	6.60 (1.776)	.56		25.30 (2.791)		.88			
	Posttest	5.90 (1.73)	.54		17.30 (3.35)		1.05			
**Group C**				.01						<.001
	Pretest	6.90 (1.73)	.54		24.30 (2.75)		.87			
	Posttest	5.40 (1.07)	.33		13.20 (4.661)		1.47			
**Group D**				<.001						<.001
	Pretest	7.10 (1.53)	.48		24.20 (2.098)		.66			
	Posttest	3.60 (.97)	.30		3.30 (1.49)		.47			

^a^ODI: Oswestry disability index.

^b^*P* values are significant at ≤.05.

**Table 3:** Intergroup comparison for pain and disability index.

Group and compared group		Pain rating		ODI^a^	
		Mean difference	*P* value	Mean difference	*P* value
**Group A**					
	Group B	–1.70	.04	–4.20	.002
	Group C	–1.20	.23	–.10	>.99
	Group D	.60	.76	9.80	.001
**Group B**					
	Group A	1.70	.04	4.20	.02
	Group C	.50	.85	4.10	.02
	Group D	2.30	.00	14.00	.001
**Group C**					
	Group A	1.20	.23	.100	.68
	Group B	–.50	.85	–4.10	.02
	Group D	1.80	.03	9.90	<.001
**Group D**					
	Group A	–.60	.76	–9.80	<.001
	Group B	–2.30	<.001	–14.00	<.001
	Group C	–1.80	.03	–9.90	<.001

^a^ODI: Oswestry disability index.

#### Low Back Disability

After 8 sessions, the pre- and posttest ODI were 25.10 (SD 3.035) and 13.10 (SD 1.85) in group A, 25.30 (SD 2.791) and 17.30 (SD 3.35) in group B, 24.30 (SD 2.75) and 13.20 (SD 4.661) in group C, and 24.20 (SD 2.098) and 3.30 (SD 1.49) in group D, respectively (Table 2). In the intergroup analysis, group D showed dominant effects on the disability index compared to groups A, B, and C, with mean differences of –9.80, –14.00, and –9.90 (all *P*<.001), respectively (Table 3).

#### Lumbar ROM

The pre- and posttest mean scores for lumbar flexion were 36.10 (SD 4.91) and 42.50 (SD 5.78) in group A, 37.20 (SD 3.43) and 38.90 (SD 2.64) in group B, 36.10 (SD 4.91) and 41.50 (SD 5.42) in group C, and 37.20 (SD 3.42) and 46.30 (SD 1.95) in group D, respectively. The pre- and posttest mean scores for right lateral lumbar flexion were 12.90 (SD 1.19) and 16.70 (SD 1.05) in group A, 13.60 (SD 1.95) and 14.30 (SD 2.35) in group B, 12.90 (SD 1.197) and 15.60 (SD 0.966) in group C, and 13.50 (SD 1.96) and 19.5 (SD 3.57) in group D, respectively. The pre- and posttest mean scores for left lateral lumbar flexion were 12.90 (SD 1.19) and 16.0 (SD 1.76) in group A, 13.60 (SD 1.96) and 14.00 (SD 1.24) in group B, 14.60 (SD 2.41) and 16.60 (SD 2.17) in group C, and 14.60 (SD 1.35) and 18.80 (SD 1.32) in group D, respectively. The pre- and posttest mean scores for lumbar extension were 8.6 (SD 1.71) and 11.60 (SD 1.84) in group A, 7.90 (SD 1.10), and 8.70 (SD 0.94) in group B, 8.20 (SD 1.686) and 10.70 (SD 1.636) in group C, and 7.90 (SD 1.37) and 13.50 (SD 0.85) in group D, respectively (Table 4).

The mean differences in forward lumbar flexion ROM for group D compared to groups A, B, and C were 3.80 (*P*=.21), 4.80 (*P*=.07), 7.40 (*P*<.001), respectively. The mean differences in right lateral lumbar flexion ROM for group D compared to groups A, B, and C were 2.80 (*P*=.04), 3.90 (*P*<.001), and 5.20 (*P*<.001), respectively. The mean differences in left lateral lumbar flexion ROM for group D compared to groups A, B, and C were 2.80 (*P*<.001), 2.20 (*P*=.02), and 4.80 (*P*<.001), respectively. The mean differences in lumbar extension ROM for group D compared to groups A, B, and C were 1.90 (*P*=.02), 2.80 (*P*<.001), and 4.80 (*P*<.001), respectively (Table 5).

**Table 4:** Intragroup comparison for lumbar range of motion.

Range of motion and evaluation		Group A			Group B			Group C			Group D		
		Mean (SD)	Mean SE	*P* value	Mean (SD)	Mean SE	*P* value	Mean (SD)	Mean SE	*P* value	Mean (SD)	Mean SE	*P* value
**Lumbar forward flexion**				<.001			.38			<.001			<.001
	Pretest	36.10 (4.91)	1.55		37.20 (3.43)	1.08		36.10 (4.91)	1.55		37.20 (3.42)	1.08	
	Posttest	42.50 (5.78)	1.83		38.90 (2.64)	0.83		41.50 (5.42)	1.71		46.30 (1.95)	0.61	
**Right lateral lumbar flexion**				<.001			.10			<.001			<.001
	Pretest	12.90 (1.19)	0.37		13.60 (1.95)	0.61		12.90 (1.197)	0.37		13.50 (1.96)	0.61	
	Posttest	16.70 (1.05)	0.33		14.30 (2.35)	0.74		15.60 (0.966)	0.3		19.5 (3.57)	1.13	
**Left lateral lumbar flexion**				<.001			.49			<.001			<.001
	Pretest	12.90 (1.19)	0.37		13.60 (1.96)	0.61		14.60 (2.41)	0.76		14.60 (1.35)	0.42	
	Posttest	16.0 (1.76)	0.27		14.00 (1.24)	0.39		16.60 (2.17)	0.68		18.80 (1.32)	0.41	
**Lumbar extension**				<.001			<.001			<.001			<.001
	Pretest	8.6 (1.71)	0.54		7.90 (1.10)	0.34		8.20 (1.686)	0.53		7.90 (1.37)	0.43	
	Posttest	11.60 (1.84)	1.55		8.70 (0.94)	0.30		10.70 (1.636)	0.51		13.50 (0.85)	0.26	

**Table 5:** Intergroup comparison for lumbar range of motion.

Groups and compared group		Forward lumbar flexion		Right lateral lumbar flexion		Left lateral lumbar flexion		Lumbar extension	
		Mean difference	*P* value	Mean difference	*P* value	Mean difference	*P* value	Mean difference	*P* value
**Group A**									
	Group B	3.60	.25	2.40	.09	2.00	.05	2.90	<.001
	Group C	1.00	.95	1.10	.69	–.600	.85	.90	.47
	Group D	–3.80	.21	–2.80	.04	–2.80	<.001	–1.90	.02
**Group B**									
	Group A	–3.60	.25	–2.40	.09	–2.00	.05	–2.90	<.001
	Group C	–2.60	.53	–1.30	.57	–2.60	<.001	–2.00	.01
	Group D	–4.80	<.001	–5.20	<.001	–4.80	<.001	–4.80	<.001
**Group C**									
	Group A	–1.00	.95	–1.10	.69	.60	.85	–.90	.47
	Group B	2.60	.53	1.30	.56	2.60	<.001	2.00	.01
	Group D	–4.80	.07	–3.90	<.001	–2.20	.02	–2.80	<.001
**Group D**									
	Group A	3.80	.21	2.80	.04	2.80	<.001	1.90	.02
	Group B	4.80	.07	3.90	<.001	2.20	.02	2.80	<.001
	Group C	7.40	<.001	5.20	<.001	4.80	<.001	4.80	<.001

## Discussion

### Principal Findings

LBP is one of the major musculoskeletal health issues prevalent among the general population. This quasi-experimental study was conducted among 40 patients with chronic LBP who were treated with VR exercises along with traditional exercises. VR exercises were found to have dominant effects in improving pain, low back disability, and lumbar ROM in the different groups, but the combination of VR games, including the Reflex Ridge and Body Ball games with back-strengthening exercise, was better than other groups.

### Comparison With Prior Work

This study was conducted among 40 patients. We hypothesized that VR exercises through the Reflex Ridge and Body Ball games could be one of the effective methods used in the clinical management of LBP and improve lumbar ROM and disability index, similar to the findings of dodge ball games in the literature [6]. Our study results favored VR exercises for improving pain, low back disability, and lumbar ROM. Park et al [30] used the Nintendo Wii exercise program for LBP and reported that exercise programs significantly improved physical function related to LBP. In health-related quality of life, the Nintendo Wii exercise program showed significant improvements in both the mental and physical health composites, but other groups showed significant improvement only in the physical health composite.

The integration of VR with physiotherapy was found to be effective for pain, ROM, disability index, and kinesiophobia. Experimental treatment with VR reduced pain and improved physical function in patients with acute and chronic pain as well [15]. In another study, a VR dodgeball intervention provided evidence of safety and feasibility and can be used to encourage spinal flexion in individuals with chronic LBP [6]. Group D reduced pain intensity compared to other groups that were treated with the Body Ball or Reflex Ridge game alone (group A: *P*=.76; group B: *P*<.001; and group C: *P*=.03), showing significant and better results than all other groups.

One of the reasons for using VR games is that it induces a postexercise hypoalgesic effect and a significant reduction in thinking of pain, which further enhances its implication in clinical studies for pain management [12]. This study correlated with our study, as the Body Ball and Reflex Ridge VR games along with exercises are intended to allow movements in the lumbar region within a virtual environment, and the involvement of participants while playing the game elicits enthusiasm and eagerness to perform activity throughout the session. In our study, low back disability index differences in the groups had *P* values <.05, showing improvement in all 4 groups. Yilmaz Yelva et al [15] stated that VR had a positive impact on pain and kinesiophobia in individuals with chronic pain. In their study among patients with subacute and chronic nonspecific LBP, virtual walking integrated with physiotherapy decreased pain and improved function in the short term. Their findings are similar to our study, but the games administered were different. Wiederhold et al [31] stated that VR as a distraction technique is effective in reducing pain intensity and discomfort with significance ranging from *P*=.05 to *P*=.001. A previous study has shown significant effects of VR exercises for improving pain, disability, and ROM but has not compared which VR exercise game is more feasible and effective [23]. In our study, the lumbar ROM—including flexion, extension, and lateral flexion on both sides—improved in all groups, but intragroup comparisons showed that group D with a combination of VR and exercise had superior effects in improving the lumbar ROM with significant pre- and posttest differences. This finding demonstrated that the VR exercises had an additive effect and led us to assume that these exercises can be an option for the treatment of LBP, similar to the effects seen in core stability exercises [32]. This emerging technology has been used for the nonpharmacological management of LBP and resulted in less use of nonsteroidal anti-inflammatory drugs. VR has been considered as an analgesic as it works based on the distraction phenomenon to decrease pain [33]. VR exercises compared to traditional exercises exert a positive impact on psychological, physiological, and rehabilitative outcomes, but there is a need trial different games to better rehabilitation programs [34].

Despite the novelty of the technique, different VR games may lead to rapid pain relief in addition to routine management strategies. Different VR games in different age groups and clinical trials are recommended for better generalization of the results.

### Limitations

The study conditions and participant characteristics may not represent the broader population of interest due to limited generalizability to other populations and settings. There was no random assignment of participants and this lack of randomization can introduce selection bias. Despite these limitations, a quasi-experimental study is valuable especially in a situation where a randomized controlled trial was not feasible due to the COVID-19 pandemic.

### Conclusion

VR exercises are effective as treatment strategies in the management of LBP. Both VR games had significant effects in improving lumbar ROM, pain intensity, and low back disability, but a combination of the Reflex Ridge and Body Ball games along with back-strengthening exercises was found to be more effective.

## Abbreviations

LBP: low back pain
ODI: Oswestry disability index
ROM: range of motion
VR: virtual reality
